# Author Correction: Automating data analysis for hydrogen/deuterium exchange mass spectrometry using data-independent acquisition methodology

**DOI:** 10.1038/s41467-024-47157-z

**Published:** 2024-04-02

**Authors:** Frantisek Filandr, Vladimir Sarpe, Shaunak Raval, D. Alex Crowder, Morgan F. Khan, Pauline Douglas, Stephen Coales, Rosa Viner, Aleem Syed, John A. Tainer, Susan P. Lees-Miller, David C. Schriemer

**Affiliations:** 1 Department of Biochemistry and Molecular Biology, University of Calgary, Calgary, AB T2N 4N1 Canada; 2 Department of Chemistry, University of Calgary, Calgary, AB T2N 4N1 Canada; 3 Trajan Scientific & Medical - Raleigh, Morrisville, NC USA; 4 Thermo Fisher Scientific, San Jose, CA USA; 5 Division of Radiation and Genome Instability, Department of Radiation Oncology, Dana-Farber Cancer Institute, Harvard Medical School, Boston, MA 02215 USA; 6 Department of Molecular and Cellular Oncology, The University of Texas MD Anderson Cancer Center, Houston, TX 77030 USA; 7 Molecular Biophysics and Integrated Bioimaging, Lawrence Berkeley National Laboratory, Berkeley, CA 94720 USA

**Keywords:** Proteome, Bioanalytical chemistry

Correction to: *Nature Communications* 10.1038/s41467-024-46610-3, published online 11 March 2024

The original version of this Article omitted the funding source for some authors: “J.A.T. and S.L.M. were funded in part by National Institutes of Health (NIH) grant P01 CA092584, and J.A.T. and A.S. were funded in part by R35 CA220430 and a Robert A.Welch Chemistry Chair (G-0010).” This has now been corrected in both the PDF and HTML versions of the Article.

